# Accurate Diet Assessment, Patient Selection, and Objective Clinical Outcome Are Critical in Untangling the Role of Diet on Pouchitis

**DOI:** 10.1093/crocol/otad079

**Published:** 2023-12-09

**Authors:** Dakota Rhys-Jones, Chu K Yao, Zaid S Ardalan

**Affiliations:** Department of Gastroenterology, Central Clinical School, Monash University and Alfred Health, Melbourne, Victoria, Australia; Department of Gastroenterology, Central Clinical School, Monash University and Alfred Health, Melbourne, Victoria, Australia; Department of Gastroenterology, Central Clinical School, Monash University and Alfred Health, Melbourne, Victoria, Australia

We read with interest the observational study reported by Barnes and colleagues,^1^ who analyzed baseline dietary intake of a cohort of patients with various inflammatory pouch disorders, and its impact on disease activity. We congratulate the authors for recruiting such a large cohort in a rare patient group. However, we believe the authors overlooked several points that are crucial when generating their study conclusions.

First, there are flaws in the dietary assessment approaches utilized in this study. Primarily, an assessment of “dietary patterns” which involves “whole-diet” analysis using statistical methods, was not performed, rather a comparison of nutritional intake.^2^ Second, the low intake of several food groups and dietary fiber in the cohort with a history of pouchitis reinforces findings of other studies.^3,4^ It should be noted that nutritional recommendations (such as for fiber) are formulated for healthy individuals with a colon. It is unclear whether these guidelines are relevant when applying them to patients with an ileoanal pouch, particularly in the context of preventing inflammatory conditions of the pouch. Perhaps involvement of a research dietitian with expertise in global assessment methods will provide insights into whether dietary patterns do have a role to play in the development or worsening of pouchitis.

Second, the clinical outcome used to define disease activity was the clinical part of the modified pouchitis disease activity index, namely the clinical PDAI (cPDAI).^5^ However, pouch-related symptoms constituting the cPDAI are not specific for the diagnosis of inflammatory disorders of the pouch and cannot distinguish flares from noninflammatory disorders of the pouch such as irritable pouch syndrome. In the instance that all the symptomatic patients had a flare, the absence of information on their risk factors for chronic pouchitis such as smoking status, age of diagnosis of ulcerative colitis, extraintestinal manifestations, and whether they were on maintenance treatment or not, makes attributing differences in rates of flare to differences in nutritional intake or lack thereof alone problematic.

Finally, nearly two-thirds of the patients had Crohn’s disease of the pouch (51%) or chronic antibiotic-refractory pouchitis, which are predominately immune-mediated processes. When trying to determine whether dietary intake independently predicts pouchitis, it is more useful to either exclude this cohort or at least have the majority of the cohort with no history of pouchitis, antibiotic-responsive pouchitis, and dependent pouchitis where the pouch microbiota has purportedly a more predominate role.

In summary, Barnes et al highlight the challenges of investigating whether dietary factors predict the development or the worsening of pouchitis, and the importance of having holistic dietary assessment methods, objective clinical outcomes, and the right cohort whose risk factors for pouchitis are well-characterized.

## References

[1] Barnes EL , Beniwal-Patel P, Deepak P, et al. Dietary patterns are not associated with disease activity among patients with inflammatory conditions of the pouch in a prospective cohort. Crohns Colitis 360 2023;5(3):otad039. 37519405 10.1093/crocol/otad039 PMC10374273

[2] Shanahan ER , McMaster JJ, Staudacher HM. Conducting research on diet–microbiome interactions: a review of current challenges, essential methodological principles, and recommendations for best practice in study design. J Hum Nutr Diet. 2021;34(4):631–644. 33639033 10.1111/jhn.12868

[3] Godny L , Maharshak N, Reshef L, et al. Fruit consumption is associated with alterations in microbial composition and lower rates of pouchitis. J Crohns Colitis 2019;13(10):1265–1272. 30828722 10.1093/ecco-jcc/jjz053

[4] Ardalan ZS , Livingstone KM, Polzella L, et al. Perceived dietary intolerances, habitual intake and diet quality of patients with an ileoanal pouch: associations with pouch phenotype (and behaviour). Clin Nutr. 2023;42(11):2095–2108. 37748240 10.1016/j.clnu.2023.07.023

[5] Shen B , Achkar J-P, Connor JT, et al. Modified pouchitis disease activity index: a simplified approach to the diagnosis of pouchitis. Dis Colon Rectum. 2003;46(6):748–753. 12794576 10.1007/s10350-004-6652-8

